# Financial team incentives improved recording of diagnoses in primary care: a quasi-experimental longitudinal follow-up study with controls

**DOI:** 10.1186/s13104-015-1602-1

**Published:** 2015-11-11

**Authors:** Tuomo Lehtovuori, Timo Kauppila, Jouko Kallio, Marko Raina, Lasse Suominen, Anna Maria Heikkinen

**Affiliations:** Espoo, Finland; Department of General Practice and Primary Healthcare, HUS, Institute of Clinical Medicine, University of Helsinki, P.O. Box 20, Tukholmankatu 8 B, 00014 Vantaa, Finland; Vantaa, Finland; Department of Oral and Maxillofacial Diseases, University of Helsinki, Espoo, Finland

**Keywords:** Financial incentive, Primary care, Group bonus

## Abstract

**Background:**

In primary care, financial incentives have usually been directed to physicians because they are thought to make the key decisions in order to change the functions of a medical organization. There are no studies regarding the impact that directing these incentives to all disciplines of the care team (e.g. group bonuses for both nurses and doctors) may have, despite the low frequency with which diagnoses were being recorded for primary care visits to doctors. This study tested the effect of offering group bonuses to the care teams.

**Methods:**

This was a retrospective quasi-experimental study with before-and-after settings and two control groups. In the intervention group, the mean percentage of visits to a doctor for which a diagnosis was recorded by each individual care team (mean team-based percentage of monthly visits to a doctor with recorded diagnoses) and simultaneously the same data was gathered from two different primary care settings where no team bonuses were applied. To study the sustainability of changes obtained with the group bonuses the respective data were derived from the electronic health record system for 2 years after the cessation of the intervention. The differences in the rate of marking diagnoses was analyzed with ANOVA and RM-ANOVA with appropriate post hoc tests, and the differences in the rate of change in marking diagnoses was analyzed with linear regression followed by t-test.

**Results:**

The proportion of doctor visits having recorded diagnoses in the teams was about 55 % before starting to use group bonuses and 90 % after this intervention. There was no such increase in control units. The effect of the intervention weakened slightly after cessation of the group bonuses.

**Conclusion:**

Group bonuses may provide a method to alter clinical practices in primary care. However, sustainability of these interventions may diminish after ceasing this type of financial incentive.

## Background

Tailored payment systems have been used in attempts to achieve policy objectives, such as improving the quality of care or recruitment to under-served areas, because the method by which physicians are paid may affect their professional practice [1, 2]. Conventionally, these financial incentives have been directed to physicians because they are thought to make the key decisions to change the functions of a medical organization [3–5]. There are ample recent studies concerning how delivering financial incentives to primary care physicians alone may alter the physicians’ behavior of and thereby the performance of the care system [6–12].

Yet, in modern multidisciplinary health care systems there are also other quite autonomous actors, such as nurses who specialize in the treatment of diabetes [4, 5, 13, 14], who may influence the functions of their organization significantly and also improve the quality of the care. Thus, also other disciplines than doctors might well be considered as objects for financial incentives in improving the quality of care. Improving the recording of diagnoses of acute and chronic diseases might theoretically serve as one of the most important targets [15–18], and would therefore be a suitable element to improve by financial incentives. The recording of diagnoses in only 40–60 % of doctor visits in the care units was deemed insufficient by the administration of the primary health care of Espoo City. A higher frequency of recorded diagnoses was deemed necessary for planning activities and to manage the resources of primary health care. This led to the present intervention and study.

The aim of the present study was to examine whether it is possible to improve clinical practice by increasing the recording of the diagnoses through the use of financial incentives to all disciplines in the care team (e.g. group bonuses). We were also interested in how enduring these changes in the frequency of to registering the diagnoses would be after cessation of payment of these group bonuses.

## Methods

This study was performed in Espoo city where in 2006 there were 230,000 inhabitants. As everywhere in Finland, primary care is non-profit and it is maintained by municipalities which fund this activity with taxes. In Espoo, there are five municipal health service areas which each contain 3–6 care teams. Altogether the number of care teams was 23. There were 6–8 doctors and 6–8 nurses per team. The precise amount of doctors and nurses varied slightly over the study period.

This is a retrospective quasi-experimental study. The executive of Espoo primary care defined the areas where improvement was desired and their goal levels at the start of 2005. Improvement of recording diagnoses of the patient charts was chosen as the main goal. In order to obtain the group bonus it was necessary for teams to record diagnoses for doctor visits at a significantly higher rate than before intervention. The proportion of monthly doctor visits having recorded diagnoses was selected as the main measure to study the effect of implementing group bonuses. In practice this meant that to get a group bonus a care team had to take care that diagnoses were recorded in more than 75 % of all doctor visits of that team. Diagnoses were recorded with ICD-10 or ICPC systems by the doctors. Both systems, Effica and Finstar, gave a similar specific place in the electric patient chart where appropriate ICPC-2 or ICD-10 diagnoses could be placed during the patient visit. Both systems assisted the GP to find a proper diagnosis code or allowed the doctor to use directly the right code for the desired diagnosis.

To commit the staff to the change in function, a multidisciplinary team contract was signed with the members of the care teams. The contract defined the rules and approaches of the functions of the teams. The team contracts were signed by all of the five service areas between 1.3.2005 and 30.5.2005, which was considered to be the time of the start of the intervention. The data was specifically derived from the electrical Effica patient chart system (Tieto LTD, Helsinki, Finland) from which the data had been reliably obtainable since 1.5.2003. No ethical approval was required because this study was made directly from the patient registry without identifying the patients. The registry keeper (the health authorities of Espoo and Vantaa) granted permission to carry on the study. The report generator provided figures for the total number of doctor visits, the number of recorded diagnoses and thus a percentage for the recording of diagnoses for each individual doctor and thereby also for the care unit per month. This allowed the calculation of a mean of these percentages for each individual care team (mean care unit-level percentage of doctor visits with marked diagnoses/care unit/month) which was thus the main measure for analysis in the present study.

The obtained data were analyzed by comparing the recording of diagnoses during similar time periods before and after the initiation of group bonuses to all nurses and doctors belonging to the care teams (intervention) in primary care in Espoo city.

As control data, we had the corresponding data on the single doctor and care team level frequencies of the recording of monthly diagnoses from two different primary care units where no similar team incentives were applied: dental primary care of Espoo and Länsimäki–Hakunila primary care health center from the neighboring city of Vantaa. Vantaa resembles Espoo in its location (neighboring Helsinki) and number of inhabitants (about 200,000) and also in other factors such as age, sex, morbidity levels, deprivation and other demographic factors as much as possible in Finland (see http://www.aluesaarjat.fi, and http://pxweb2.stat.fi/database/StatFin/databasetree_fi.asp) and therefore we have used Vantaa as a control in our former studies of primary care, too [19].

From Espoo dental care the data were analogously obtainable from 1.5.2003 (also using Effica patient chart system, Tieto LTD, Helsinki, Finland). The data of the combined Länsimäki–Hakunila health center were obtained from Finstar patient chart system (Logica LTD, Helsinki, Finland). To get reliable data from Finstar-system the report generator requires precise pre-identification of the doctor under study at a given time point and it is therefore not able to produce continuous monthly data throughout the whole system, as is the Effica system. Therefore the busiest month of the year (November) was chosen as the control data and comparisons between controls were made by using this single time period.

The Effect of the intervention on the mean team-based percentage of monthly doctor visits with recorded diagnoses was monitored for a 2 years time period before intervention and 1.5 years after it. Since the group bonus system was altered in such a way that after 2010, e.g. during 2011 and 2012, the recording of diagnoses did not produce a financial bonus for the care team, we collected the data of the same parameter from the teams existing in Espoo health center in November 2011 and 2012 after the cessation of the intervention. In this way we hoped to have some information about how enduring the changes obtained with the team bonus combined with team contract were.

The within-care team variation in Espoo primary care, Espoo dental care and Länsimäki–Hakunila primary care were analyzed by using the mean team-based percentage of monthly doctor visits with recorded diagnoses over the whole study period. The comparisons were then performed by using One Way Repeated Measures ANOVA with suitable corrections (Bonferroni) for multiple comparisons when following the development of the studied units as a function of time. One way ANOVA on Ranks followed by Dunns’ test was used to compare Espoo primary care with the control units at corresponding time points. The rate of change in diagnosis marking was analyzed with regression analysis followed by t-test (GLM procedure of Sigma Plot 10.0 Statistical Software, Systat Software Inc.,Richmond, CA, USA) [20, 21].

## Results

**Fig. 1:**
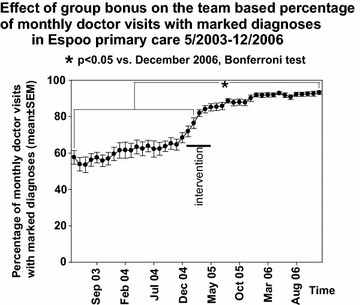
Mean team-based percentage of monthly doctor visits with marked diagnoses 2003–2006. Means and SEM are presented

**Fig. 2:**
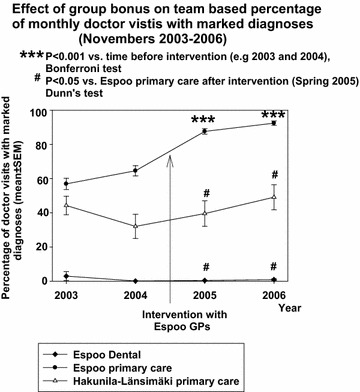
Mean team-based percentage of monthly doctor visits with marked diagnoses in intervention group Espoo primary care and in controls. Means and SEM of Novembers 2003–2006 are presented

In Espoo primary care, the mean number of monthly visits in office-hour services of primary care doctors was about 18,000 in 2003–2006 and in primary care EDs the mean number of doctor visits per month was about 4000 in the same period suggesting a total number of about 22,000 monthly doctor visits in the whole public primary care system of Espoo city. In the complementary private sector primary care the mean number of monthly doctor visits was about 4500 in 2006. The mean team-based percentage of monthly doctor visits with recorded diagnoses increased from about 55 to 90 % after application of group bonuses in Espoo primary care (one way repeated measures analysis of variance P < 0.001; Figs. 1, 2). There was already a slight increase (0.79 ± 0.12 %/month, mean ± SEM) in the rate of marking diagnoses before the intervention in Espoo primary care. However, during the six first months of intervention this rate doubled statistically significantly to 1.65 ± 0.39 %/month (P = 0.005). After the six first months of intervention, the increase in the rate plateaued to the level of 0.31 ± 0.07 %/month, which was statistically significantly less than before the intervention (P = 0.002) or during the first six months of the intervention (P < 0.001).

Simultaneously, when the group bonus system was associated with an increased proportion of monthly doctor visits having recorded diagnoses in care teams of Espoo primary care there were no increases in the same parameters in either of the controls (Fig. 2). The team-based mean percentage in recording diagnoses did not differ statistically significantly in Espoo primary care and Länsimäki–Hakunila, but in both these units this frequency was statistically significantly higher than in Espoo dental care in the beginning of the follow up period in 2003 (one way ANOVA on ranks P < 0.001, Dunns’ test, P < 0.05). However, after the intervention in 2005 and 2006 the mean frequency of recording diagnoses was higher in Espoo primary care teams than in either of the teams of the control units (one way ANOVA on ranks P < 0.001). The rate of marking diagnoses increased in in the Espoo primary care 12.95 ± 1.13 %/year during the follow up time. This was statistically significantly more (P < 0.001) than in either of the controls: in Hakunila–Länsimäki primary care (Vantaa) this rate (mean ± SEM) was 1.99 ± 3.05 %/year and in Espoo dental primary care it was negative −0.53 ± 0.53 %/year. In practice this means that the controls did not differ from each other statistically significantly and that there was no change in the rate of marking diagnoses in either of the controls during the follow up.

**Fig. 3:**
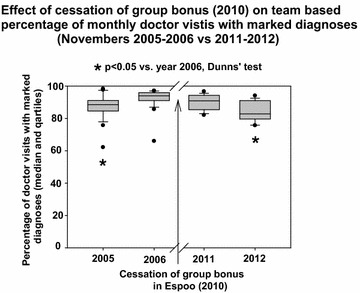
Mean team-based percentage monthly doctor visits with marked diagnoses in intervention group Espoo primary care just after the beginning of paying the group bonuses (years 2005 and 2006) and after their cessation (2010) in years 2011 and 2012. Means and SEM of Novembers are presented

The mean team-based percentage of monthly doctor visits with recorded diagnoses started to decrease within 2 years of cessation of the team bonus (2010; one way analysis of variance on Ranks, P < 0001, Fig. 3). During the first full year after intervention (2005–2006) the rate of recording diagnoses was still increasing (4.82 ± 2.07 %/year) but it was statistically significantly different (P < 0.001) from the respective rate during the first full year after cessation of the bonuses (2011–2012) when this parameter decreased to −19.70 ± 6.56 %/year.

In 2005, 28.9 % of staff of the care teams received group bonuses. Mean annual group bonus varied between 205.15 and 601.77 €/person depending on the care team. In the second year of intervention (2006), 49.1 % of the staff of these teams received group bonuses worth 592.45–768.75 €/person.

## Discussion

A financial incentive, introducing group bonus with team contracts, improved the recording of diagnoses in the patient charts. Neither in the dental unit of Espoo, which here represented the part of the same primary care organization where no intervention was applied (e.g. different specialization in the same organization; internal local control) nor in Länsimäki–Hakunila, which here represented a neighboring organization with the same specialization (e.g. somatic primary care in different organization: external peer control) were there similar increases in the studied parameters during the same time period. Although differences across computer systems exist and even these systems may be predictors of care quality outcomes [22] this does not explain the results of our study because we had an internal control (primary dental care of Espoo) which used the same computerized patient chart system as the intervened Espoo primary care.

Before the intervention it was expected that the low level of recording diagnoses was due to different administration and management cultures in different health service areas, lack of permanent doctors and deficiency in tutor services. However, financial incentives combined with team contracts with the care teams seemed to overcome these putative hindrances to proper recording of patient data. The present finding with group bonuses is in line with a former study where financial incentives to GPs increased diagnosis making and recording of certain diseases [7, 9]. Altogether, our work with other recent studies suggests that financial incentives may be used to alter behavior of physicians towards improving the quality of care [8, 23–26]. Furthermore, financial incentives may promote other important values in primary care such as reduction of inequalities in the delivery of clinical care related to area deprivation [6].

Whether rewarding staff with financial incentives leads to real quality improvement in care is still an open question [27]. Evidence about the effectiveness of this kind of intervention at the population level appear contradictory as in a large scale former study there was no evidence of lower mortality rates of the population [12] but in another study of similar scale there was evidence for reduction in emergency hospital admissions after a pay-for performance intervention for GPs [10]. Altogether, this means that one must choose carefully the measures of improved care: a hard end point like mortality is not easily affected [15]. Furthermore, improved recording of diabetes and related parameters does not automatically guarantee better care of the disease itself [28] and improvements in quality aspects of care do not automatically result in better outcomes of care [8]. Nevertheless, recorded diagnoses make it possible to analyze data further and possibly find areas of improvement in the local quality of care [17]. Several questions, such as how reliable and valid the data obtained with the present intervention is, have to be answered before giving any recommendations about the usefulness of group bonuses in improving clinical practices.

There appeared to be units where frequencies of recording diagnoses in doctor visits decreased after cessation of payment of the group bonuses. This latter is in line with former reports suggesting that those parameters in quality work which are not sustained with financial incentives to GPs may even weaken, if improving other parameters is rewarded [6, 29]. Yet, partial withdrawal of financial incentives did not largely hamper results obtained with this type of intervention to GPs [11]. Altogether, financial incentives have been reported to provide large initial gains which, however, diminished over time [8]. This holds true for financial incentives directed to patients, too: only that part of behavior which is paid for is improved [30]. Thus, the eventual consequences of the behavior change are not necessarily in line with the original intention of the intervention driven with economic incentives [8, 30]. The present follow-up time (2 years) is relatively short to answer the question of what will eventually happen in the long term when the group bonuses are totally withdrawn. Yet, the level of recording diagnoses in 2012 was still clearly superior to the years before the intervention (2003 and 2004). Altogether, the present finding is thus in line with a hypothesis that financial incentives are effective primers in interventions of primary care [6, 8], even when group incentives to the care team are applied.

## Conclusion

Group bonuses may provide a method to improve clinical practices in primary care. Yet the putative desired effects obtained with these financial incentives may slowly start to erode if these bonuses are withdrawn.
